# Prediction of Urban Street Public Space Art Design Indicators Based on Deep Convolutional Neural Network

**DOI:** 10.1155/2022/5508623

**Published:** 2022-05-11

**Authors:** Shanshan Yu, Hao Wang

**Affiliations:** ^1^School of Landscape Architecture, Nanjing Forestry University, Nanjing, Jiangsu 210037, China; ^2^School of Architecture and Civil Engineering, Xiamen University, Xiamen, Fujian 361005, China

## Abstract

This paper analyzes and studies the structure and parameters of the VGGNet network model and selects the most commonly used and efficient VGG-16 as the prototype of the improved model. A multiscale sampling layer is added at the end of the VGG-16 convolution part so that the model can input images of any size for training and testing while reducing the number of neurons in the fully connected layer. This improves the training speed of the model under the premise of ensuring the accuracy. This paper uses multisource street spatial data combined with geographic information spatial analysis technology to measure and evaluate the spatial quality of streets in the main urban area. From the three dimensions of vitality, safety, and greenness of urban street space quality, a systematic structure for evaluation and analysis of street space quality is constructed. Street vitality includes eight index factors: entrance and exit density, street furniture density, street sketch density, street characteristic landscape density, POI density, POI diversity, commercial POI ratio, and street population density. There are five index factors: degree, roadside parking occupancy ratio, traffic signal system density, sidewalk width proportion, and street facility density. We use ArcGIS to build an index factor information database for statistical analysis and visualization. According to the natural discontinuous point classification method, the safety level of urban street public space is divided into five grades. The sample size of the first four grades has a small fluctuation range. The sample sizes are 153, 172, 153, and 158, respectively, accounting for 21%, 23%, 21%, and 21% of the total street samples, of which the first two grades occupy a total of 44%, so 44% of the streets in the main urban area have a low-quality level of street space. Level 5 has a sample of 102 streets, accounting for 14%, with an average street space quality value of 0.43.

## 1. Introduction

Under the impact of intelligent concepts and technologies, the complexity of cities has become increasingly prominent, people's psychological activities have been subtly affected, and a series of social forms have been updated and transformed. The development momentum led by information technology has further formed the differentiation between physical space and virtual space [1]. The new data environment enables us to observe microscopic physical space and fine social space. Traditional urban space is not enough to meet the new behavior of users [2]. Ways and living habits, such as the emergence of house culture, have made people's requirements for urban public space and public life higher, and the sharing of information in the era of big data has also increased the radiation range of public space [3]. However, the current research on the construction of intelligent urban space is in the ascendant, and the update research on the urban space design method is urgently needed. With the advancement of human technology, street design has gradually improved from the traditional motor-driven and standardized technical street design paradigm to an innovative design of street livability and interaction. Some foreign cities have begun to rediscover the value of street space and have launched various types of “street design manuals,” redefining the connotation and use of street design and triggering the city's most potential public space street [4].

As an important type of urban space, urban street space will also show different characteristics of the times with the changes of urban development and technological culture [5–7]. Combined with the influence factors of intelligent environment on urban street space, this paper analyzes the characteristics of contemporary urban street space and provides a scientific reference for urban street space design. At present, the construction stage of smart cities has entered the transition stage from physical integration to public service integration [8]. Smart methods are emerging at various stages of urban planning, but most of them focus on the macrolevel of cities, and there is little applied research on the microlevel. Therefore, based on the existing research, this paper focuses on the application of intelligent means in urban streets. With the rise of my country's smart city construction, papers on related issues have increased rapidly and have just begun to form an upsurge. However, most of the research content is based on a macroperspective, which cannot meet the current needs of smart city construction. In the research on the theme of “smart city public space,” there is some mention of “street space,” but there is a lack of systematic research [9]. The backwardness of theory will hinder the progress of practice. Therefore, the research on urban street space design based on the smart city construction index system in this paper is of great significance. In the project research, in addition to sorting out new digital information technology, the characteristics of smart cities are also implemented into the design of contemporary urban street space, exploring new ideas for the design of physical and nonmaterial space in the street under the background of the times.

This paper studies the deep convolutional neural network VGGNet, explores the characteristics of the model, and analyzes its structure and parameters; then, on the basis of the VGG-16 network model, the VGG-MSL network model is improved and optimized, and the multiscale network model is added. The sampling layer allows the network to input samples of any size and reduces the width of the fully connected layer to improve the training efficiency of the model. In order to fully understand the composition of street space and the characteristics of the factors affecting the quality of street space, this study conducted a detailed investigation and data sorting on the basic situation of street space in the main urban area. The content of this paper mainly focuses on three aspects: establishing the measurement model of vitality, safety, and greenness; building a street quality evaluation system; and visualizing the evaluation results. We select a number of indicators from the three dimensions of vitality, safety, and greenness to obtain their factor weights through the analytic hierarchy process, establish a measurement model of street vitality, urban street public space safety, and street greenness, and obtain street vitality, urban street public space safety, and street greenness. The measurement results of safety degree and street green degree are used as the comprehensive index value of street quality, and the comprehensive evaluation system of street quality is established by the entropy value method.

## 2. Related Work

The convolutional neural network evolved based on the early network structure Neocognitron, which replaces the simple cells and complex cells with the convolutional layers and pooling layers in the convolutional neural network, which is a multilayer neural network. Its main feature is that it can fully consider the invariance of scaling, rotation, and translation of samples in space, which improves the convergence and generalization capabilities of the model.

Related scholars have achieved high classification accuracy by building a CNN network model LeNet-5 and using it for the recognition of handwritten digits [10]. The model is obtained through a gradient backpropagation training algorithm. The input handwritten characters are converted into many feature maps through the alternate connection of multiple convolutional layers and downsampling layers. Finally, the obtained sample feature expression is processed through the fully connected layer. Among them, various convolution kernels in the convolution layer excite the local area information of the bottom layer to a higher level through the local receptive field and obtain a more abstract feature expression through layer-by-layer transmission [11]. Its success has caused a boom in the application of convolutional neural networks, and the research of convolutional neural networks in the fields of speech recognition, target detection, semantic segmentation, and face recognition has gradually been carried out [12]. With the continuous development of computer technology, including software and hardware, and the arrival of the era of big data deep learning, it is possible to train and implement deeper and more complex convolutional neural networks.

Related scholars have conducted a series of analyses on the practice of smart cities in Europe [13]. Through technical methods such as data collation and model establishment, the relevant technical strategies of smart city construction are summarized and applied to the planning and construction project of Chongming Smart Ecological Island [14]. The big data and smart city research team has made systematic research in related fields from big data application in urban planning, urban and rural planning, and design based on big data to urban and rural special planning and urban and rural evaluation management based on big data [15]. The researchers worked on planning support systems and microsimulation of urban systems and successfully analyzed the spatiotemporal distribution characteristics of commuting trips using bus IC card swiping data, mobile phone data, and Weibo data [16].

In recent years, the research enthusiasm of domestic and foreign scholars for urban street space has not declined, but most of them focus on three aspects: one is the planning and environmental design research corresponding to people's needs and behaviors; the other is the needs and design of urban development space; the third is the inheritance and development of traditional urban planning [17]. With the accelerated construction of urbanization and the continuous development of information technology, the application level of urban informatization has been continuously improved, and new spatial forms have been derived from urban street space, which has gradually attracted the attention of relevant scholars [18].

In the related research on intelligent street space design, scholars studied the influence of commercial space and interface characteristics on pedestrians' staying activities through the method of questionnaire survey [19]. Relevant scholars have given indicators for the prediction and evaluation of urban street vitality, but due to the difficulty in obtaining indicator data, only targeted research has been done [20]. The researchers comprehensively evaluated the plot vitality index through street accessibility, construction intensity, architectural form, and functional mixing and used GIS data to verify the inference [21]. Relevant scholars have studied the application of big data in the design scale of urban public space from the perspective of urban streets, the design process, and the prospect of smart streets based on virtual technologies such as big data and cloud computing and conceived the big data ecosystem of street design. Relevant scholars took the Beijing Urban Laboratory as a carrier to quantify the development of streets from an interdisciplinary approach and conducted a visual analysis and research on the spatial-related data of urban streets [22]. In the field of practice, the design of street space is gradually breaking the subjective consciousness-oriented design method, and the acquisition of street space information is not limited to official data. With the popularization of digital information technology and network technology, various data open platforms have been established one after another.

## 3. Methods

### 3.1. Deep Convolutional Neural Network VGGNet Model

**Figure 1:**
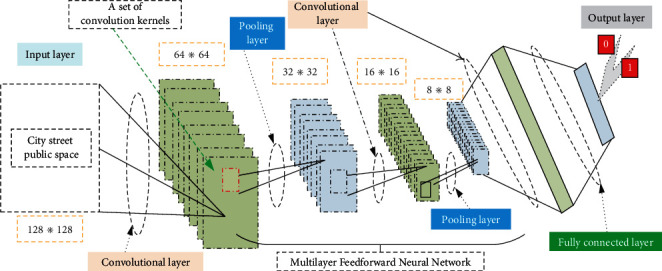
Deep convolutional neural network architecture.

DCNN is a multilayer feed-forward neural network, where each layer uses a set of convolution kernels to perform multiple transformations. Convolution operations help to extract useful features from locally correlated data, assigning the output of convolution kernels to nonlinear processing units; this nonlinearity produces different activation patterns for different responses, thus facilitating learning semantic differences in samples. DCNNs are specifically designed to process samples, so neurons in each layer are organized in three dimensions, height, width, and depth, just like pixels in a sample distinguish different color values. The important properties of DCNN are hierarchical learning, automatic feature extraction, multitask processing, and weight sharing, which are mainly composed of convolutional layers, excitation layers, pooling layers, and fully connected layers.  Figure 1 shows the architecture of a deep convolutional neural network.

The image can be divided into regions for consideration, and then each neuron is connected to a local region of the input. The extent of this connectivity is called the receptive field of the neuron and is equivalent to the size of the filter.

It is important to emphasize this asymmetry when dealing with spatial and depth dimensions. Connections are local in space, but always global over the entire depth of the input.

The training of DCNN includes two processes of forward propagation and backward propagation. During forward propagation, the data is propagated backward from the input layer to the output layer layer by layer, and the activation values of each layer of the network are calculated until the last layer; during backward propagation, the gradient is calculated according to the error, and the gradient goes from the last layer to the layer by layer. When the gradients of all layers are calculated, the network parameters are updated by the gradient descent method, and the parameters are continuously updated by calculating the partial derivatives of the loss function relative to the network parameters.

A training set *X* consists of *m* samples, where *x*(*l*) is a single sample and *y*(*l*) is the label corresponding to the sample. The loss function for a single sample is as follows:

(1) $$\begin{array}{c} J\left(W,-b|x,y\right)=0.5b{\left[y-h_{W,b}\left(-x\right)\right]}^{2}. \end{array}$$

Then, the loss function for the entire dataset is

(2) $$\begin{array}{c} J\left(W,-b\right)=\frac{\lambda }{m-1}\prod_{i=0}^{m-1}{\left[y^{\left(i\right)}-h_{W,b}x^{\left(i\right)}\right]}^{2}-\prod_{l=1}^{n_{l}}\prod_{j=1}^{s_{l}}\prod_{i=1}^{n_{l}}W^{2}{}_{ji,l}. \end{array}$$

The first term on the right side of the equation calculates the total error term, which represents the sum of the errors of all samples; the second term is the regularization term, which is used to control the magnitude of weight decay during network training. The role of the parameter *b* is to control the trade-off between the regularization term and the mean squared error term.

After the overall loss function is obtained, the gradient is then calculated for parameter update. The formula for gradient descent to update the weights *W* and bias *b* is as follows:

(3) $$\begin{array}{c} W_{ij,l}=\alpha W_{\left(i-1\right)j,l}-\frac{\partial J\left(W,b\right)}{\partial W_{ij,l}},\\ b_{i,l}=\alpha b_{\left(i-1\right),l}-\frac{\partial J\left(W,b\right)}{\partial b_{i,l}}. \end{array}$$

VGGNet is a DCNN completed by Oxford University and Google Deep Mind. It can be regarded as a deepened version of AlexNet. It has successfully constructed several CNNs with a depth of 16 to 19 layers, such as VGG-16 and VGG-19. The most used is VGG-16.  Table 1 is the main comparison of AlexNet and VGGNet-16. The VGGNet network model has the following main contributions.

First, VGG verifies the influence of network depth on the accuracy rate and verifies that there is a relationship between network depth and model performance by constructing neural networks with different layers.

Second, VGG uses a 3 × 3 convolution kernel to replace the large convolution kernel used in the previous network, which not only expands the depth of the network but also reduces the convolution kernel. Features are very precise. In addition, if we adopt the stacked small convolution kernel, the effect is much stronger than that of the large convolution kernel. This is because the multilayer nonlinear layer can realize the effect of deepening the network, thereby enhancing the generalization ability. Compared with AlexNet, VGGNet seems to have more parameters, but its convergence is also very easy, which is the effect of a deeper network and smaller convolution kernel.

Third, the importance of parameter initialization is verified. By controlling the relevant experimental conditions, the accuracy of various initialization situations is measured. The experimental results show that there is a close relationship between the initialization of the parameters and the training results. The root cause of this problem is that there are still some problems in the model itself, resulting in locally optimal solutions during training.

### 3.2. VGGNet Network Structure and Parameter Configuration

The VGGNet network model structure can be divided into six categories, which are represented by A, A-LRN, B, C, D, and E, respectively. The network structure and parameter configuration are shown in Table 2.

As can be seen from the table, the structure of the VGGNet model is very simple and clear, consisting of a 5-segment convolutional part, a 3-layer fully connected layer, and a SoftMax output layer. On the whole, the convolution part is usually closely connected with the max-pooling layer, and the activation units of the hidden layer all use the ReLU function. The number of layers in each convolutional part is roughly between 2 and 4, and the 5-segment convolutional part adopts the 64-128-256-512-512 mode, where the number can be considered as how many features are extracted by the convolutional layer. At the same time, the convolution kernels and pooling layers of the same size are used in various models. The final output layer is the SoftMax layer, which can realize multiclassification, draw corresponding conclusions, and make judgments by analyzing the probabilities of various types. For example, in a sample classification scenario, the input sample can be probabilistically discriminated. If the sample contains a target, the output is the correct category; otherwise, the wrong category is output.

### 3.3. Improvement Ideas

The size of the input samples of the deep convolutional neural network model VGGNet is fixed. Only when samples of any size are converted to this size can they be input into the model. There are some disadvantages of this manual operation method.

One is that when the sample data is massive, it takes a lot of data preprocessing time; the other is that cropping or distortion will change the size of the sample, resulting in some important loss, and will also change the shape of the sample, which is difficult to match with reality. The third is that if the shape or size of the sample to be subjected to the related operation changes relatively greatly, the preprocessing operation or network training must be performed again to adapt to the changed sample to ensure the increase of the sample recognition accuracy. If the last three fully connected layers of VGGNet are changed to convolutional layers and VGGNet is improved into a fully convolutional neural network, the limitation of fully connected layers does not exist. The model can input samples of any size, thereby improving the training efficiency of the model in the testing phase.

Although this scheme can solve the limitation of the fixed size of input samples, there is no fully connected layer in the entire network model, which affects the performance of the network to a certain extent. Previously, our understanding of the role of fully connected layers was limited to “classification”; that is, fully connected layers can map what has been learned to the sample label space.

The existence or not of the connected layer will directly affect the transfer learning results of the network. Experiments show that transfer learning results tend to be more ideal for networks with connected hierarchies. That is to say, the fully connected layer should be regarded as a “firewall,” which can provide a certain degree of guarantee for achieving a good transfer learning effect.

From this, we can know that completely replacing the fully connected layer with the convolutional layer will lose the performance of the network model, especially when the model is subjected to transfer learning. Therefore, this paper adopts the method of adding a multiscale sampling layer to avoid the full connection layer to the sample input size. At the same time, on the one hand, the model can input samples of any size, and on the other hand, the fully connected layer is retained, which ensures the generalization performance of the model during transfer learning.

However, the number of parameters of the fully connected layer accounts for 80% of the entire network parameters. This parameter redundancy will greatly increase the training complexity and cause overfitting. Therefore, this paper further considers reducing the width of the fully connected layer. The number of neurons in the connection layer can reduce the number of parameters, further improve the training efficiency of the model, and try to avoid the occurrence of overfitting.

### 3.4. Methods of Multiscale Sampling

In order to adapt the network to input samples of various sizes, this paper proposes to add a multiscale pooling layer after the sixth convolutional part. The multiscale pooling layer mentioned here refers to the diversity choice of sampling size and stride. By pairing the sampling window with the input samples one by one, the sampling scale or step size can be adjusted according to the input so as to produce the effect that the size of the input does not affect the number of sampling windows so that the number of sampling windows can be adjusted. For example, after the convolutional layer, the size of a feature function spectrum is *r* × *s*, and three sampling sizes *ξ* and stride Φ are used for maximum sampling. The specific calculation formula is as follows:

(4) $$\begin{array}{c} \xi =\begin{array}{ccc} \left(r-1\right)•s & \left(\frac{r}{2-1}\right)•\frac{s}{2} & \left(\frac{r}{3-1}\right)•\frac{s}{3} \end{array},\\ \Phi =\begin{array}{ccc} \left(r-1\right)•\left(s-1\right) & \left(\frac{r}{2-1}\right)•\frac{\left(s-1\right)}{2} & \left(\frac{r}{3-1}\right)•\frac{\left(s-1\right)}{3} \end{array},\\ y_{j,1}=\text{Max}\left(r•s•l•x_{i}\right),\\ y_{j,2}=\text{Max}\left[\left(\frac{r}{2}\right)•\left(\frac{s}{2}\right)•\left(\frac{l}{2}\right)•x_{i}\right],\\ y_{j,3}=\text{Max}\left[\left(\frac{r}{3}\right)•\left(\frac{s}{3}\right)•\left(\frac{l}{3}\right)•x_{i}\right]. \end{array}$$

The obtained output feature matrix is expanded in the order of columns and cascaded in sequence to obtain a feature column vector of fixed size; multiscale fixed sampling allows input samples of any size and any aspect ratio. Therefore, we can use multiple scales of the input samples to extract multiple graph features at different scales.

### 3.5. VGG-MSL Network Structure Design

**Figure 2:**
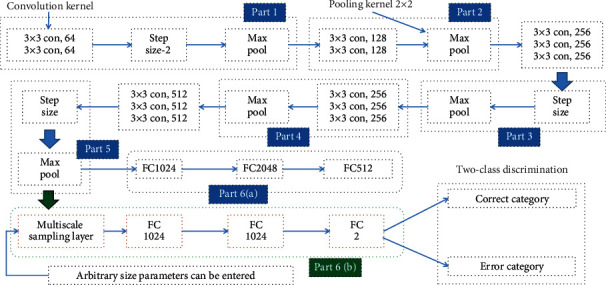
VGG-MSL network model design.

Due to the property of full-layer connection limitation, the size of all sample inputs of the deep convolutional neural network model VGG-16 is fixed to 224 × 224. In order to input samples of any size into the network model, this paper proposes a multiscale fixed sampling scheme. The multiscale sampling layer is placed after all the convolution parts, and then the fully connected layer is connected. Considering the huge amount of parameters in the fully connected layer, the resulting increase in the amount of computation and the requirement for computing power on the computing platform are also getting higher and higher, so we hope to remove the neural network in the fully connected layer on the basis of ensuring the accuracy, thereby reducing the amount of parameters and establishing an efficient model. After analyzing the VGG-16 research, this paper proposes a design improvement as shown in Figure 2.

The improved multiscale fixed sampling VGG-MSL network model structure is also divided into 6 parts. In part 6, a multiscale sampling layer is added, and the width of the first two fully connected layers is reduced by three-quarters; that is, 1024 neurons are used, and the output of the last layer is 2, corresponding to the category and the noncategory.

In general, the VGG-MSL network model is improved on the basis of the VGG-16 model. The core is that a multiscale sampling layer is added after the fifth convolution part, so that the network can input parameters of any size, and three-quarters of the neurons are subtracted from the first two fully connected layers in the sixth part. The number of parameters is reduced, which helps to improve the training efficiency, and the last layer is two-class discrimination.

### 3.6. VGG-MSL Network Model Parameter Selection

The convolutional neural network model VGG-MSL model designed in this paper uses max-pooling. On the whole, the calculation of max-pooling is not difficult, and the efficiency is high. A convolution kernel is equivalent to a feature extractor, and the convolution kernels in different environments complete the extraction of each feature, respectively. For example, the *a* convolution kernel is responsible for extracting the “color” feature, and the *b* convolution kernel is responsible for extracting the “texture” feature. However, those data that are useless or even hinder the extraction of specific features should be discarded. Therefore, max-pooling can be regarded as a simple and effective pooling method.

The activation function is in a key position in the process of realizing network conduction. It can not only play the role of adjusting the parameter value but also ensure that the network can realize the expression of more levels and more kinds of content by adding nonlinear factors. It is convenient to solve more difficult nonlinear problems.

The feature of the Sigmoid-type function is that it can increase the signal in the middle, but it has the opposite effect on the signals on both sides and can weaken its signal. It is relatively easy to saturate. When this function is used, the process can terminate the error propagation, which will bring great problems to the later training of deep networks. Different from the Sigmoid-type function, the ReLU function can converge quickly, can suppress unilateral signals, and has a wider excitation boundary. In addition, it also has the characteristics of sparseness and activity.

In summary, the model VGG-MSL designed in this paper adopts the ReLU activation function. Using this activation function, although there is an inherent part of the same defect as the Sigmoid function, that is, when the input value is very large or very small, it is prone to saturation, it has a significant advantage that its output is 0-centered.

### 3.7. Indicator Factor Weights

The differences in the indicators of street vitality, safety, and greenness result in different effects on quality. Therefore, the weight of each indicator should be reasonably determined, and then the value of each element indicator and the weight should be superimposed. A comprehensive result is obtained to judge the quality of street space. In this paper, the AHP analytic hierarchy process is used to determine the weight of each index factor through statistical methods. As the street type changes, the validity of the index factors also changes. In this study, the weights of the street vitality, safety, and greenness index factors are calculated, respectively, as shown in Table 3. In terms of weight calculation, the index factor hierarchy is determined by the AHP. In order to increase the objectivity of the weight determination, the “entropy value method” is used when calculating the weights of the three dimensions of street vitality, safety, and greenness.

## 4. Results and Analysis

### 4.1. Experimental Environment

The core of the experimental environment in this paper is the TensorFlow deep learning framework, the CUDA parallel computing architecture, and the cuDNN GPU acceleration library. The developers of the TensorFlow deep learning framework are from the Google Brain team. They use a large number of functions and algorithms related to machine learning and neural networks to enable them to explore machine learning and deep neural networks. CUDA (Compute Unified Device Architecture) is a model created by NVIDIA that can parallelize computing and programming, and the model can perform computations while programming operations occur. NVIDIA cuDNN is the company's special GPU-accelerated library that can effectively speed up the training of neural networks.

The experimental environment is based on Windows system, and the code is written in Python language. The specific environment configuration is shown in Table 4. This configuration environment is used in the experiments in subsequent chapters of this paper.

### 4.2. Prediction and Evaluation of the Safety Degree of Art Design in Urban Street Public Space

Traffic signal systems exist to ensure safe passage. When there are only street signs and no traffic signal system, people will have concerns about the safety of crossing the street, especially when crossing the street in a large amount of dense traffic, which increases the danger of crossing the street.

This sense of danger mostly comes from pedestrians' psychological distrust of street safety, and the traffic signal system increases this level of trust. By default, people follow traffic signal rules to ensure their own safety. Therefore, this study uses the density of street traffic signal system as one of the index factors to measure the degree of street safety.

The data is divided into 5 grades by the natural discontinuous point method, and the range from grade 1 to grade 5 is 0.00–0.07, 0.08–0.19, 0.20–0.34, 0.35–0.56, and 0.57–1.00, respectively. Through data analysis, the traffic signal system density of comprehensive streets and traffic streets is significantly higher than that of other streets, while some living streets do not have traffic signal systems. Whether or not they have a traffic signal system has become a watershed for the safety of living streets. Through statistical analysis, there are 197 streets without a traffic signal system, accounting for 27% of the total number of streets, of which there are 186 living streets, accounting for 94% of the total number of streets without a traffic signal system. Therefore, this study believes that the lack of traffic signal system in living streets is the biggest and most urgent problem to be solved in the existing streets.

**Figure 3:**
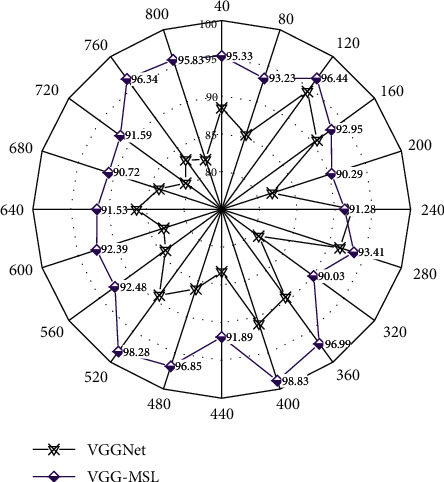
Distribution of public space safety degree in urban streets under different data samples.

The public space safety degree of urban streets shows a different distribution from the vitality of the streets. It reflects the uneven distribution of living streets in the public space safety of urban streets.  Figure 3 shows the distribution of public space safety in urban streets under different data samples.

**Figure 4:**
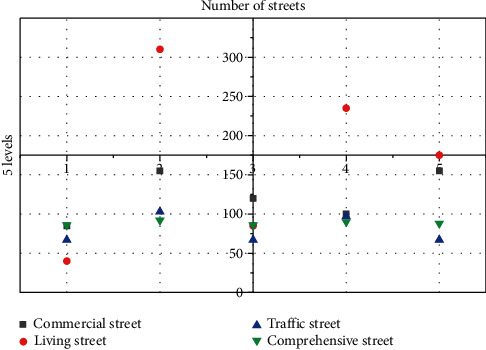
Distribution of the number of different types of streets in each grade.

Living streets include 294 street samples in the first three grades, accounting for 94% of the total living streets, while 72% and 76% of the sample sizes of commercial streets and comprehensive streets are in the two high grades of 4 and 5, respectively. It can be shown that the safety of the public space of living city streets is mostly at a low level. At the same time, most of the public space safety of commercial streets and comprehensive urban streets is at a high level of street level. There are 404 street samples in the two high-level street vitality levels 4 and 5, accounting for 55% of the total sample. It can be seen that about 45% of the urban streets in the main urban area have a low level of public space safety.  Figure 4 shows the distribution of the number of different types of streets in each level of public space safety in urban streets.

### 4.3. Prediction and Evaluation of the Vitality of Urban Street Public Space Art Design

**Figure 5:**
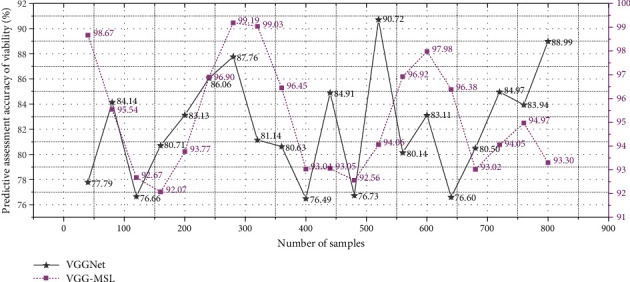
Predictive assessment of the vitality of streets without entrances and exits.

After processing the exit density along the street, it is found that the distribution trend is obvious. Among them, there are 164 streets with the density of motor vehicle entrance and exit along the main road being 0. The branch road serves the convenient connection inside and outside the plot, which shows that more than half of the living streets need to increase the entrances and exits of motor vehicles along the street, so as to improve the communication ability between the street enclosed plot and the external space and reflect the internal support of the plot from the side. The predicted evaluation of the vitality of the street without entrance and exit is shown in Figure 5.

Street space furniture and facilities are the basis for users to carry out behavior activities, and the convenience of facilities has an important impact on the use and evaluation of street space. A large number of studies have shown that setting up rest seats in public spaces can make people stay for a long time, thus greatly increasing the utilization rate of the space. In addition, most of people's continuous and social activities take place next to walls, shop windows, and other facilities on the sidewalk, including flower stands, seats, and vending machines. The above street facilities not only play their own specific functions and are convenient for people use but also, at the same time, increase the richness of the street space. Generally speaking, the facilities in the street space include municipal facilities, road traffic facilities, art landscapes, and event service facilities. The street facilities discussed in this study focus on street furniture facilities. Street furniture is a basic public service facility, and its lack reflects the lack of public service facilities and the poor environmental quality of public space.

**Figure 6:**
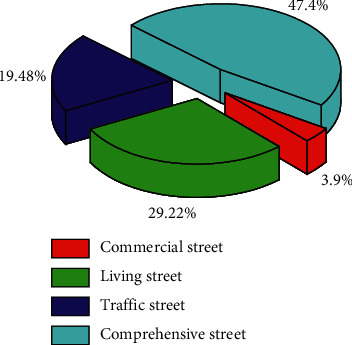
Street composition of street furniture index factors.

It also shows obvious distribution characteristics among street furniture owners. In Figure 6, comprehensive streets account for most of the proportion of street furniture owners. Traffic streets and comprehensive streets belong to arterial roads, accounting for 68% of the proportion of street furniture owners. The furniture configuration of urban arterial roads is significantly higher than that of other roads, and among all streets, 3 streets have the highest street furniture density.

If the characteristic landscape along the street is regarded as a large-scale urban landmark, the street sketch can be regarded as a detailed urban accessory, and the overall effect of the street space is reflected by a large amount of detailed art. In the case of small differences in themes, some details can better reflect the cultural quality and aesthetic taste of a city.

Street sketches are the point elements of urban spiritual and cultural construction and reflect the cultural heritage of a city in subtle ways. Street sketches not only enrich the street space but also carry and disseminate culture. Street sketches are a key element in bringing the street space environment to life.

The higher the density of street sketches, the higher the vitality of the street. The ranges from level 1 to level 5 are 0.00–0.04, 0.05–0.12, 0.13–0.26, 0.27–0.45, and 0.46–1.00, respectively. The distribution of street sketches has a certain correlation with the distribution of street furniture and is generally distributed in streets connecting street squares and street green spaces. Most of the locations with street furniture are also equipped with street pieces, but compared with the lack of a large number of pieces of street furniture, there are still a large number of pieces set up independently in places such as street green belts, resulting in a difference in density between the two. There are 235 more streets with street furniture than streets with street furniture, and 132 streets with both are similar to the number of streets with only street furniture, which shows that the number of pieces of street furniture is partially related to street furniture, but the distribution of street furniture is more for broad.

The five levels of data are divided by the natural discontinuous point method. The range from level 1 to level 5 is 0.00–0.54, 0.55–0.77, 0.78–0.88, 0.89–0.94, and 0.95–1.00, respectively. Comparing and analyzing the POI diversity of different types of streets, the average value of commercial streets and traffic streets is the same and smaller than that of living streets and comprehensive streets; the standard deviation is generally small, the standard deviation of traffic street data is relatively the largest, and the diversity of street POI diversity is relatively large.

**Figure 7:**
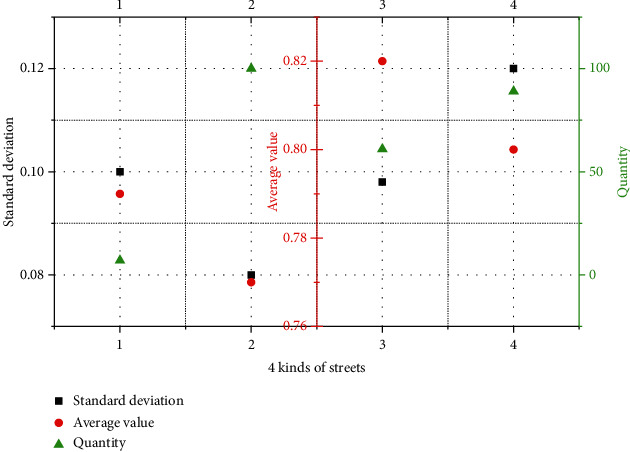
POI diversity level V data composition.

In each grade, grade V has 247 streets, and living streets in Figure 7 occupy the largest proportion in the highest-grade POI diversity index, which shows that living streets have a higher degree of business diversity. According to Figure 7, this study believes that the diversity level of street POIs decreases in order from living streets, commercial streets, comprehensive streets, and traffic streets, and living streets have a richer and more stable business pattern than the other three.

### 4.4. Prediction and Evaluation of the Greenness of Urban Street Public Space Art Design

There are 141 streets in the main urban area with street-facing interfaces adjacent to street green spaces. Such street green spaces include street garden green spaces, square green spaces, park green spaces, landscape green spaces, and protective green spaces. Different from the single form of green belt, street green space exists in various forms, and all have relatively good organization forms. Street green space not only enriches the street space environment but also echoes with street trees and green belts to form an urban green space system.

Temporary greening facilities often appear in the main urban area. Compared with the monotonous green of the green belt, temporary greening facilities are the finishing touch to make the street greening colorful. As a city with a heavy cultural load, its architectural style tends to be mature and stable, and colorful scenes are rarely seen in the street space. Temporary greening facilities are mostly composed of combined flower beds, which are composed of flowers of various colors and become a bright spot to break the dullness in the street space.

**Figure 8:**
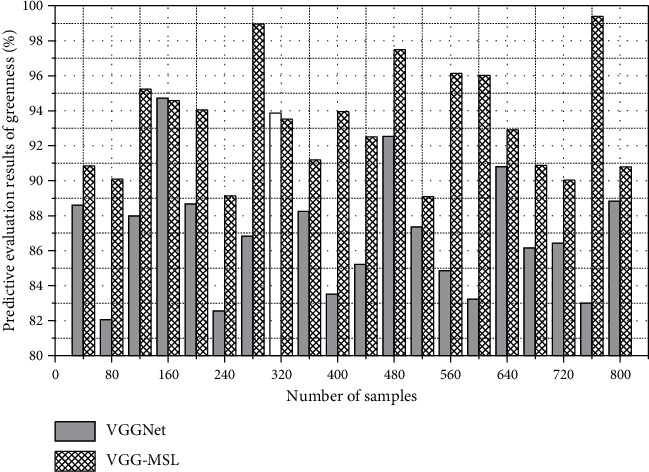
Prediction and evaluation results of the greenness of urban street public space art design.

Temporary greening facilities in the main urban area are mostly found in road intersections, missing parts of street green belts, street lamp hanging flower baskets, and other open spaces in the street. Although the number is relatively small, they are essential to the street space in the main urban area, and temporary greening facilities have their own advantages. The unique advantage is that the temporary greening facilities can be flexibly changed with seasons and festival themes.  Figure 8 shows the prediction and evaluation results of the greenness of urban street public space art design.

## 5. Conclusion

Aiming at the problem that the convolutional neural network model is limited by the input sample size and the training process is time-consuming, this paper proposes a new multiscale sampling VGG-MSL network model based on VGGNet-16. On the basis of ensuring the accuracy, the model structure is simplified, and a multiscale sampling layer is added. It is experimentally verified that the model reduces the training speed and can input samples of any size to it. This paper constructs a street space quality evaluation system with three dimensions of street vitality, safety, and greenness and visualizes the index factors to obtain the distribution characteristics of street space quality in each dimension. We conduct a comprehensive analysis of the street quality in the main urban area and summarize three circles of street quality distribution in the study area. High-quality agglomeration areas are mainly concentrated in the southern part of the regional center, occupying 15% of the total area, and about one-sixth of the study area is in the gradient of high-quality streets. The gradient of the medium-quality agglomeration area accounts for about 64% of the total area, and the gradient of the low-quality agglomeration area accounts for 21% of the total area; that is, about 60% of the street quality in the study area is in the middle-quality agglomeration area, and the overall street quality of the main urban area is at an average level. Through the research and analysis of this paper, it can be known that the vitality, safety, and greenness of street space are indispensable, and the three are not separated and independent from each other but are complementary and integrated. In the process of street design, it is necessary to use dynamic thinking to achieve a balance between vitality, safety, and greenness, so as to optimize the overall street space quality.

## Figures and Tables

**Table 1:** Comparison of AlexNet and VGGNet.

	Structure	Step size	Parameter (M)	Convolution kernel
AlexNet	8 layers: 5 convolutional layers and 3 fully connected layers	6	54	5 *∗* 5
VGGNet-16	16 layers: 13 convolutional layers and 3 fully connected layers	2	116	3 *∗* 3

**Table 2:** The structure and parameter configuration of each VGGNet model.

8 weight layers	10 weight layers	12 weight layers	14 weight layers	16 weight layers
Input layer				
conv3-64	conv3-64	conv3-128	conv3-128 conv3-64	conv3-128 conv3-128
Max pool				
conv3-512 conv3-512	conv3-512 conv3-512	conv3-512 conv3-512	conv3-512 conv3-512 conv1-512	conv3-512 conv3-512 conv3-512 conv3-512
Max pool				
FC-1024				
FC-1024				
FC-512				
SoftMax				

**Table 3:** Index factor weights.

Index factor	Commercial street	Living street	Traffic street	Comprehensive street
Degree of isolation	0.06	0.12	0.05	0.03
On-street parking occupancy ratio	0.04	0.11	0.02	0.01
Traffic signal system density	0.09	0.15	0.17	0.05
Sidewalk width	0.12	0.17	0.09	0.02
Density of crossing facilities	0.22	0.31	0.13	0.21
Characteristic landscape density along the street	0.02	0.10	0.03	0.01
Street sketch density	0.14	0.15	0.03	0.07
POI diversity	0.02	0.11	0.08	0.09
POI density	0.13	0.17	0.09	0.05
Proportion of commercial POIs	0.22	0.14	0.08	0.07
Number of green belts	0.21	0.32	0.06	0.05
Street tree density	0.04	0.16	0.03	0.11
Proportion of the length of street green space	0.14	0.19	0.23	0.30
Density of temporary greening facilities	0.03	0.16	0.19	0.33
Street shade ratio	0.03	0.16	0.09	0.02

**Table 4:** Experimental environment.

Programming language	Hardware environment	Development environment	Development platform	Operating system
MATLAB	CPU: AMD 2700X GPU: NVIDIA GEFORCE GTX 2070 Memory: 32G	Spyder	TensorFlow 1.4.0 CUDA 10.1 cuDNN 7.0	Windows 10 ultimate

## Data Availability

The data used to support the findings of this study are available from the corresponding author upon request.
